# Unlocking the secret life of blue mussels: Exploring connectivity in the Skagerrak through biophysical modeling and population genomics

**DOI:** 10.1111/eva.13704

**Published:** 2024-05-20

**Authors:** Malin Gustafsson, Åsa Strand, Ane T. Laugen, Jon Albretsen, Carl André, Göran Broström, Per Erik Jorde, Halvor Knutsen, Olga Ortega‐Martinez, Marte Sodeland, Malin Waern, Anna‐Lisa Wrange, Pierre De Wit

**Affiliations:** ^1^ Environmental Intelligence IVL Swedish Environmental Research Institute Gothenburg Sweden; ^2^ Environmental Intelligence IVL Swedish Environmental Research Institute Fiskebäckskil Sweden; ^3^ Department of Ecology Swedish University of Agricultural Sciences‐SLU Uppsala Sweden; ^4^ Centre for Coastal Research‐CCR, Department of Natural Sciences University of Agder Kristiansand Norway; ^5^ Institute of Marine Research, Flødevigen His Norway; ^6^ Department of Marine Sciences University of Gothenburg. Tjärnö Marine Laboratory Strömstad Sweden; ^7^ Department of Marine Sciences University of Gothenburg Gothenburg Sweden; ^8^ Leibniz‐Institute for Baltic Sea Research Warnemünde Rostock Germany; ^9^ Department of Biological and Environmental Sciences University of Gothenburg Gothenburg Sweden

**Keywords:** bivalves, dispersal modeling, management, population genetics, population structure

## Abstract

Knowledge of functional dispersal barriers in the marine environment can be used to inform a wide variety of management actions, such as marine spatial planning, restoration efforts, fisheries regulations, and invasive species management. Locations and causes of dispersal barriers can be studied through various methods, including movement tracking, biophysical modeling, demographic models, and genetics. Combining methods illustrating potential dispersal, such as biophysical modeling, with realized dispersal through, e.g., genetic connectivity estimates, provides particularly useful information for teasing apart potential causes of observed barriers. In this study, we focus on blue mussels (*Mytilus edulis*) in the Skagerrak—a marginal sea connected to the North Sea in Northern Europe—and combine biophysical models of larval dispersal with genomic data to infer locations and causes of dispersal barriers in the area. Results from both methods agree; patterns of ocean currents are a major structuring factor in the area. We find a complex pattern of source‐sink dynamics with several dispersal barriers and show that some areas can be isolated despite an overall high dispersal capability. Finally, we translate our finding into management advice that can be used to sustainably manage this ecologically and economically important species in the future.

## 1 INTRODUCTION

Increasing evidence showing small‐scale patterns of genetic differentiation has led to the recent realization that marine dispersal barriers are more prevalent than previously thought (Selkoe et al., 2008). Marine barriers to dispersal can be broadly classified into three categories: First, genetic gradients (or rapid shifts in genetic composition) can occur when populations on either side of an environmental gradient are locally adapted to the environmental conditions, so that dispersing individuals have a strongly reduced fitness in the new environment (DeFaveri et al., 2013); Second, physical barriers to dispersal, such as prevailing currents or seafloor topography, can restrict the movement of organisms with limited mobility, such as free‐drifting larvae or shallow‐water benthic organisms (Kinlan & Gaines, 2003); and third, genomic reproductive barriers generated by historical separations, leading to hybrid inviability and preventing gene mixing in secondary contact zones (Abbott et al., 2013). In many instances, natural marine systems feature combinations of these three categories, making it difficult to distinguish the relative contributions of environmental, physical, and historical barriers (Bierne et al., 2011).

Integrating ocean current data with population genetic data enables identification of dispersal barriers within the marine environment and the study of their main causes (Selkoe et al., 2008). Estimating the potential spread of larvae and the connectivity within and among geographic areas can be achieved using oceanographic modeling (Goodwin et al., 2019; Puckett et al., 2014). A common practice is to couple a hydrodynamical ocean model, such as ROMS (Regional Ocean Modeling System, http://myroms.org) or NEMO (Nucleus for European Modelling of the Ocean, https://www.nemo‐ocean.eu), to a Lagrangian particle‐drift model, e.g., CMS (Connectivity Modeling System, https://github.com/beatrixparis/connectivity‐modeling‐system), LTRANS (Larval TRANSport Lagrangian model, https://northweb.hpl.umces.edu//LTRANS.htm), LADiM (Lagrangian Advection and Diffusion Model, https://github.com/bjornaa/ladim1) or OpenDrift (https://opendrift.github.io) (Defne et al., 2016; Narváez et al., 2012; Zhang et al., 2016). Such modeling techniques can be used to simulate pathways and origins of invasive species (Laugen et al., 2015), for tracking oil spills (Röhrs et al., 2018) or for tracking fish egg/larvae from their spawning grounds (Huserbråten et al., 2018). Furthermore, the modeling can provide valuable insights for management and conservation, e.g., by providing knowledge of potential connectivity within and between populations aiding in the adaptation of the size and location of protected areas (Fulton et al., 2015; Jonsson et al., 2016). Information about the main causes of dispersal barriers can be gained by comparing locations of inferred dispersal barriers from oceanographic models and population genetic data. If long‐term water currents are the main determinant influencing connectivity patterns, then one would expect congruence between models and data, given enough time for genetic divergence to develop. However, if other factors (such as historical separations, local adaptation, alternative dispersal mechanisms, or effects of human activities) influence the genetic composition of the population, discrepancies between water current and genetic data would be expected. For instance, when combining genetic and oceanographic modeling methods to analyze the genetic structure of blue mussels in Scandinavia, Stuckas et al. (2017) found that a lack of connectivity could not fully explain the lack of introgression along the Baltic Sea transition zone, which is characterized by a strong salinity gradient. Instead, they concluded that differences in environmental selection pressures or genomic incompatibilities must contribute to the genetic structure.

In marine coastal invertebrates with long‐lived pelagic larvae, dispersal amounts, and distances can in principle both be very high. However, larval vertical swimming behavior (such as diel vertical migrations) can reduce dispersal and increase near‐shore retention of larvae (North et al., 2008), thus influencing source‐sink dynamics along coastlines (Kinlan & Gaines, 2003). Invertebrate larvae can control their vertical movement and thereby which water masses they are transported in (Genin et al., 2005; Knights et al., 2006; Shanks & Brink, 2005). However, the extent to which larval behavior interacts with vertical mixing processes in weakly swimming invertebrates is location‐specific and still relatively unknown (McIntyre et al., 2021; Weinstock et al., 2018), limiting our understanding of the ability of larvae to avoid being swept offshore (meaning certain death). Better knowledge of the actual spread of larvae and connectivity between geographic areas is important for future strategic planning of restoration projects and the identification of areas worthy of protection, where areas which contribute strongly to the export or import of larvae from larger areas, will be of a higher importance.

Blue mussels (*Mytilus* species complex) are considered keystone species in many coastal ecosystems and have for the past decades become well known for their complex interactions between historical separation, environmental gradients, and larval dispersal. Along the North Atlantic coast, three closely related species of blue mussels (*Mytilus edulis*, *Mytilus galloprovincialis*, and *Mytilus trossulus*) have all gone through intraspecific vicariance events, followed by secondary contact and intermixing (Michalek et al., 2016). In addition, the three species can interbreed and form viable hybrids in certain areas, which for example has led a hybrid population of *M. trossulus* × *M. edulis* to adapt to and colonize the low‐salinity waters of the Baltic Sea (Kijewski et al., 2006; Knöbel et al., 2021; Riginos & Cunningham, 2005; Stuckas et al., 2009), and hybrids of *M. galloprovincialis* × *M. edulis* to adapt to local conditions inside harbors in France (Simon et al., 2019). The result of all of these processes is a complex mosaic consisting of three lineages of *M. edulis*, two lineages of *M. galloprovincialis* and two lineages of *M. trossulus* in the North Atlantic (Wenne et al., 2020). However, in recent years *Mytilus*‐beds have been reported to be in decline throughout the North Atlantic (Baden et al., 2021). Consequently, there is a growing interest in restoring mussel beds, with conservation measures such as stock enhancements on the increase (Puente‐Rodríguez et al., 2015; Temmink et al., 2022). In these activities, knowledge of population structures, local recruitment patterns, and dispersal of larvae are of great value to ensure a good genetic basis for the conservation of source and sink populations and to maintain a good recruitment base and spread of new individuals. Yet, this information is currently lacking in many places, including Scandinavia, hence limiting the possibilities of assessing the impact of restoration and aquaculture activities on the mussel populations.

In this study, we investigated connectivity patterns of blue mussels in the Skagerrak, a marginal sea connecting the North Sea and Baltic Sea, through a combination of population genomic data analysis and biophysical modeling, with the aim of identifying barriers to gene flow and larval source/sink dynamics. To our knowledge, this is the first time that high‐density genetic data has been combined with biophysical transport models to infer larval mussel transport on small geographic scales. We then compare the modeling and genetic data to identify key dispersal barrier locations in the Skagerrak and provide management advice for aquaculture and restoration efforts in the area. Our results indicate that barriers to gene flow are prevalent in structuring the genetic diversity, even in a coastal marine organism with long‐lived pelagic larval stages, highlighting that this aspect should be considered in spatial planning and restoration efforts with the aim to protect local populations.

## 2 MATERIALS AND METHODS

**FIGURE 1:**
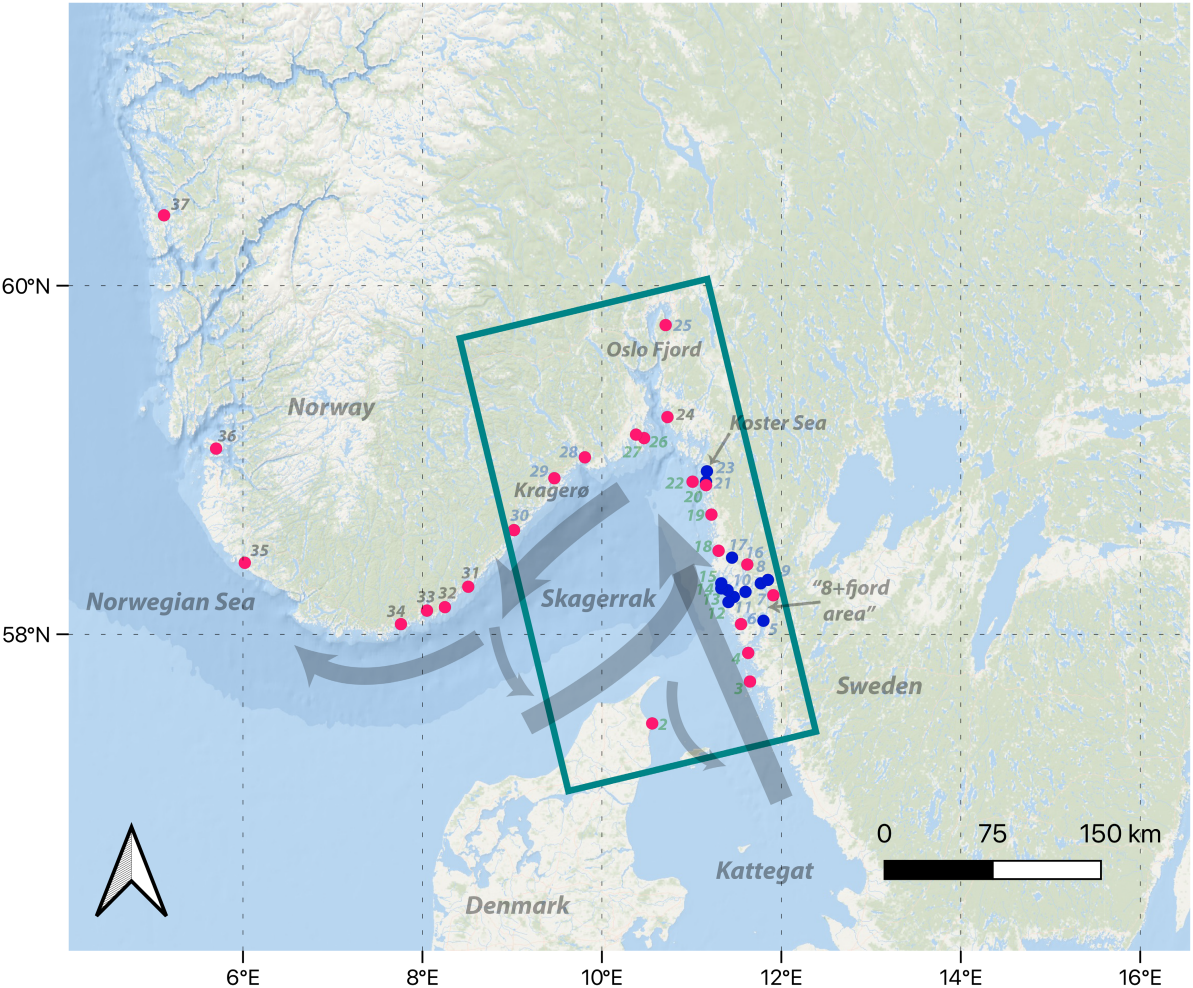
Overview map of the study area with the oceanographic modeling domain indicated by the rectangle. Study sites are denoted with circles (red circles within the rectangle are locations for which both genomic sampling and oceanographic modeling were performed, blue circles were only used in the modeling. Red circles outside of the rectangle were sampled for genomic data only). Site ID numbers are also noted, in green (over water) for outer archipelago locations, blue (over land) for inner archipelago locations, and in grey for genetics‐only sites. Main currents in the area are denoted with shaded arrows.

We modeled larval transport throughout the geographical area (Figure 1) using a combination of the hydrodynamical model ROMS and the biophysical trajectory model OpenDrift, parameterized for blue mussel larvae. For 28 sites known to contain large blue mussel beds from previous surveys (see below), outgoing and incoming larval transport was evaluated in order to infer larger‐scale patterns affecting connectivity and source‐sink dynamics. We then conducted a high‐density geographic sampling effort along the Skagerrak coast (sampling 16 of the sites used in the modeling, roughly every 20 km, with additional sites outside of the study area included for reference) for examining genome‐wide patterns of gene flow, using a 2b‐RAD genotyping approach. The genome‐wide data allowed us to filter out interfering patterns caused by introgression from divergent *Mytilus* lineages and only focus on small‐scale differentiation in the evolutionary lineage of *M. edulis* predominant in the Skagerrak.

### 2.1 Study area

The Skagerrak (Figure 1) connects the North Sea to the Kattegat and the Baltic Sea and is surrounded by the countries of Norway, Sweden, and Denmark. Predominant surface water input into the area includes the Jutland current moving water from the west coast of Denmark into the southern Skagerrak and the Baltic surface current bringing low‐salinity water from the Baltic Sea in the south northward along the Swedish coast through Kattegat. As the water masses mix, a stratification develops with the less dense Baltic water on top. During summer months, this stratification further strengthens due to the warming of the surface water, while during spring and fall deep mixing events occur regularly due to storms, bringing nutrient‐rich water to the surface. This water then circulates counterclockwise in the Skagerrak and eventually exits into the Norwegian Sea along the Norwegian coastline in the northwest (predominant currents are shown with arrows in Figure 1; Christensen et al., 2018). The area is also characterized by semi‐diurnal tides of low magnitude (ca. 30 cm), with surface water levels driven more by wind patterns than by tides, and regular upwelling events along the coast.

### 2.2 Site selection

Calculations of dispersal and connectivity were carried out for 28 locations in Sweden, Norway, and Denmark (red and blue dots inside rectangle inset in Figure 1; Table S1, from here‐on referred to as “the modeling domain”). These locations were selected in two subsequent steps. First, locations were selected evenly within the modeling domain, where *Mytilus* were found at a depth of 0.5–1 m. Second, locations identified in previous surveys (Laugen et al., 2023) to be among the best‐preserved mussel beds in the area (“category 1” beds) were added, as they have the potential to act as strong sources of larvae throughout the region, and thus constitute potential priority habitats for protection.

To assess the genetic diversity and population structures, individuals of *Mytilus* spp. were collected from 17 locations along the Skagerrak coast, along with eight sites outside of the modeling domain on the west coast of Norway (red dots in Figure 1) as well as one reference site on the Baltic Sea coast of Finland (Table S1). The genetics sites were selected to be roughly equidistant from each other, following the entire Scandinavian coastline of the Skagerrak, while at the same time provide data from reference populations from outside of the study area, as it is known that the genetic background of blue mussels is highly complex. All but one of the Skagerrak sampling locations were also included in the oceanographic modeling.

### 2.3 Hydrodynamic transport modeling

To investigate dispersal patterns of *Mytilus* larvae in the Skagerrak, larval transport was modeled using the three‐dimensional, free‐surface, hydrostatic, primitive equation model ROMS (Regional Ocean Modeling System) (Shchepetkin & McWilliams, 2005) in combination with the Lagrangian particle tracking model OpenDrift (Dagestad et al., 2018). ROMS is a numerical model generally used for simulating ocean circulation and water properties (see e.g., Neveu et al., 2016; Sen et al., 2022; Wekerle et al., 2020). In this study, ROMS was utilized on a 160 m × 160 m model grid with 35 vertical topography‐following levels shown in Figure 1. The bathymetry applied came from different sources: For the Norwegian coastal zone, bathymetry data with approximately 50 m × 50 m resolution were downloaded from the online data source, http://www.norgedigitalt.no, established by the Norwegian Mapping Authority, the Hydrographic service. For the Swedish and more central Skagerrak waters, we retrieved bathymetric data from the European Marine Observation and Data Network (EMODnet, see https://emodnet.ec.europa.eu). However, due to restrictions in the precision and resolution of the Swedish bathymetry, we performed manual adjustments of depth and coastline using datasets for bathymetry along Swedish coast (Albertsson et al., 2006) and land mask derived from National Land Cover Database (NMD) for Sweden (Shchepetkin & McWilliams, 2005).

Tides from the TPXO7.2 global tidal analysis (Egbert & Erofeeva, 2002) were included along the open boundaries together with daily averaged water level, salinity, temperature, and currents from the Baltic Sea Physics Reanalysis using NEMO provided by the EU Copernicus Marine Service Information (CMEMS), Marine Data Store (https://doi.org/10.48670/moi‐00013). The same NEMO model data was used to initialize the 160 m‐model with a start date of 2017‐01‐01. Daily river flow rates from 11 Swedish rivers were provided from SMHI's (Swedish Meteorological and Hydrological Institute) hydrological E‐HYPE (European Hydrological Predictions for the Environment) model (Donnelly et al., 2016), while similar data for 90 Norwegian rivers were based on data from NVE (Norwegian Water Resources and Energy Directorate). Atmospheric forcing was provided by AROME MetCoOp (Meteorological Co‐operation on Operational Numerical Weather Prediction) 2.5 km, the main forecasting system at the Norwegian Meteorological Institute (Müller et al., 2017).

OpenDrift is an open‐source software package for modeling the trajectories and fate of objects or substances adrift in the ocean or in the atmosphere. It is a stand‐alone python script (Dagestad et al., 2018) which can simulate the effects of winds, currents, waves, and turbulence on the movement of particles, and it can also incorporate additional forcing fields, such as sea surface temperature and salinity. For the purpose of this study, Open Drift was run utilizing hourly currents, salinity, and temperature produced from ROMS.

Reference values for hydrological and meteorological conditions in the model were taken from 2017, as this year was identified as the most typical year in terms of temperature and precipitation within the decade 2010–2019, compared to the long‐term average from 1961 to 2000. Additionally, sea surface temperatures during the summer of 2017 were found to be within the normal range, based on the average values from 2001 to 2015. Similarly, salinity levels in the Skagerrak region during the same period were also determined to be within typical ranges, as reported by Wesslander et al. (2018).

The North Atlantic Oscillation (NAO) Index was 0.23 for 2017 with a monthly SD of 0.98 (https://www.ncei.noaa.gov/access/monitoring/nao/) which is less than the average deviation from zero of the NAO Index for the period 1950–2022. Egg/larvae particles were released from each location and first tracked forward in time to see potential sink areas. Thereafter, the particles were released again from the same locations but this time they were traced back in time, to identify potential source regions.

To simulate the dispersal of eggs and larvae as accurately as possible, various parameters that may impact the spread were taken into consideration, such as the timing of gamete release, the size of the larvae, and the depth at which they drift (as outlined in Table 1). *Mytilus* spp. reproduce by releasing eggs and sperm into the water column, where fertilization occurs and free‐swimming larvae develop. As *Mytilus* spp. in the North Atlantic can reproduce over a 4‐month period, we ensured that at least one larva was released per location per hour throughout the entire period, for a total of 3000 larvae per location. For the purpose of investigating relative spread and connectivity, larvae were released at an even rate per location, although it is important to note that in reality, the actual number of gametes/larvae released varies over time and between locations. Factors such as predation and other sources of mortality were excluded from the model, as the goal was not to assess the actual number of larvae spread from each site. Additionally, the assumption was made that the larvae themselves were weak swimmers (e.g., Metaxas, 2001) and thus unable to affect their destination (passive drift), as supported by the findings of Weinstock et al. (2018) and Bonicelli et al. (2016).

**TABLE 1:** Input parameters used in the OpenDrift model for *Mytilus* larvae, based on abiotic conditions from 2017.

Parameter	Value	Reference
Release periods of larvae to be traced forward in time (spawning)	15 May—14 July: 75%	Corell (2012), Gabaev (2015), personal observation
	15 July—14 August: 25%	
Release periods of larvae to be traced backward in time (spawning +30 days)	15 June—14 August: 75%	A temperature (*x*) versus development time until settlement (*y*) model was developed based on data from Beaumont and Budd (1982), Sprung (1984), Pechenik et al. (1990), Galley et al. (2010) and Bayne (2017). A logarithmic curve was used, *y* = −28.06ln(*x*) + 101.32, *R* ^2^ = 0.7545. The model was used to calculate the average development time (*N* days) until settlement for all modeled sites based on modeled temperature data at the sites
	15 August—14 September: 25%	
Time from fertilized egg to larvae	1–2 days	Sprung (1984)
Egg size	78–85 μm	De Schweinitz and Lutz (1976), Sprung (1984), Widdows (1991)
Larval size	100–120 μm early larvae stage (D‐stage)	De Schweinitz and Lutz (1976), Sprung (1984), Widdows (1991)
	285–300 μm late larvae stage (Pediveliger)	
Time from release to settling	27–33 days	Beaumont and Budd (1982), Sprung (1984), Pechenik et al. (1990), Galley et al. (2010), Bayne (2017)
Larval drift depth (assuming neutral buoyancy for the salinity at the average drift depth)	40% 0–10 m	Raby et al. (1994), Dobretsov and Miron (2001), Corell (2012), Gabaev (2015)
	40% 10–20 m	
	20% 20–30 m	
Depth of mussel beds	70% 0.5 m	Meijerbom (2019)
	10% 2 m	
	10% 4 m	
	10% 6 m	

### 2.4 Connectivity

Gametes and larvae were released into the water column from the 28 modeled sites during the time of spawning. Following their release, these modeled larvae remained adrift in the pelagic environment for a minimum of 27 days. After this initial period, larvae had the opportunity to settle if they encountered an area with a water depth less than 10 m between days 27 and 33. However, as there is limited information on when settling is most likely to occur during this 7‐day period, the following probability calculation was employed: The study area was divided into a grid with a resolution of 0.015°, and the position of each larva during the settling period was determined. Then, the percentage of time each larva spent in different grid cells with a depth less than 10 m was calculated. For example, if a larva spent 50% of the settling period within a certain grid cell and 10% of the time within another, the first grid cell was assigned a 50% probability, and the second grid cell a 10% probability, that the larva would settle there. Once this was done for all larvae, the potential settling for each grid cell was summed. This method allowed for the estimation of the probability of settling at specific sites or in different areas (sink regions) based on the amount of time each larva spent in these areas during the settling period. To calculate potential source sites or regions the same method as described above was used but instead of releasing the larvae at the time of spawning the larvae were released from the 28 modeling sites during the settlement period and then traced backward in time for 27 days.

To estimate the connectivity between the different study sites (Table S1), we computed the potential larval transport from each site to all other sites using the method described above. Additionally, we performed a reverse calculation, determining the potential larval transport to each site from all other sites, to obtain estimates of connectivity in both directions. Finally, to facilitate a meaningful comparison between the connectivity calculations with the results from the genetic analyses (see below), we interpolated the connectivity values among locations to a finer resolution of 0.01° for the entire model domain, using the Kriging method.

To study how the location of *Mytilus* beds affects connectivity, the model sites were divided into two categories (outer/inner archipelago) according to their geographic location. Outer archipelago locations were defined as sites where larvae can easily access open water and larger ocean currents, whereas inner archipelago sites were defined as sites where large land bodies restrict larval access to open water. We tested the effect of location in the archipelago on the following response variables: (1) the number of sites to which each site contributed larvae, (2) the number of sites from which each site received larvae, (3) the total number of larvae that each site contributed to other sites, (4) the total number of larvae that each site received, (5) the proportion of larvae that were locally retained within a site. As none of the response variables followed the assumption of normality of errors, we fitted generalized models with either Poisson errors (responses 1–4) or binomial errors (response 5). The proportion of retained larvae was fitted as a two‐vector response variable of successes (number of larvae retained within a site) and failures (number of larvae that fail to settle locally). Location type (inner or outer archipelago) was fitted as a categorical predictor in all models. Due to a high degree of overdispersion, all models were fitted with quasi‐Poisson or quasi‐binomial distribution to ensure more conservative hypothesis testing. The results are presented in the text as estimated differences ±SE between inner and outer archipelago, together with the test statistics and corresponding *p*‐values.

### 2.5 Genetics

Mussels of 45–55 mm length, assumed to largely represent a single age class, were collected from the 26 selected sites. A mantle tissue sample was taken from each mussel and placed in 95% ethanol until DNA extractions. DNA was extracted using a Qiagen DNeasy Blood & Tissue kit, following the standard protocol, including the optional step of adding 4 μL RNase A (100 mg/mL) for 2 min at the end of the lysis step. DNA integrity and concentrations were determined by gel electrophoresis and QuBit DNA BR assays, respectively. Six individuals were extracted twice, as technical replicates. Reduced representation (2b‐RAD) libraries were prepared according to a modified version of the protocol designed by Wang et al. (2012), available at https://github.com/DeWitP/Mytilus. Final DNA concentrations were measured using QuBit DNA HS assays, after which the barcoded libraries were pooled equimolarly (60–95 libraries per pool) and sent to the National Genomics Infrastructure SNP&SEQ Technology Platform at Uppsala University, Sweden for sequencing using Illumina NovaSeq 6000 SP flow cells (one pool/flow cell) with 50 bp paired‐end read output. Quality of the raw sequence data was assessed using fastqc (https://www.bioinformatics.babraham.ac.uk/projects/fastqc/).

PCR duplicates were removed from the read pair data using a degenerate tag sequence added during library preparation, and the remaining data were subsequently trimmed to only keep the 36‐base 2b‐RAD fragments. The reverse sequence from each read pair was discarded as the targeted sequence fragments were shorter than the read length, meaning that the entire fragments were contained in the forward sequence. Trimmed files were further filtered for base quality, excluding any read with less than 100% called bases with *Q* > 20 using fastq_quality_filter from the fastx toolkit (http://hannonlab.cshl.edu/fastx_toolkit/). Reads were then mapped against the *M. galloprovincialis* genome sequence (Gerdol et al., 2020) (mg10.scaffolds.fa; available at: https://denovo.cnag.cat/mussel_data) using bowtie2 with default parameters, while discarding all non‐ or multiple‐aligning reads. As a reference for *M. galloprovincialis*, raw sequence reads from Gerdol et al. (2020; Files ERR2715051, 1 and 2, downloaded from https://www.ebi.ac.uk/ena/browser/view/PRJEB24883) were also mapped to the reference in the same way, after quality filtering of the raw data. The reference individual used to generate the assembly was a female from the Atlantic *M. galloprovincialis* lineage, which is known to contain introgressed *M. edulis* DNA (Diz & Skibinski, 2023). The quality of the alignments was assessed with ANGSD version 0.933 (Korneliussen et al., 2014) using the ‐doQsDist 1 and ‐doDepth 1 options, after which quality score and sequencing depth distributions among all mussel individuals were plotted with the plotQC.R script from the ngsTools package (Fumagalli et al., 2014) in R (version 3.5.1: R Core Team, 2021). This was done on a subset of the 10 first scaffolds of the genome assembly (ca. 9 MB) for which at least 1 read had been mapped in a minimum of 50% of the individuals. Individuals with more than 3 SDs lower coverage than the mean (estimated by the fraction of loci with sequencing depth ≥5 reads) were discarded from further analysis. The Identity‐By‐State (IBS) distance matrix generated by ANGSD was hierarchically clustered and examined for differences among technical replicates, after which replicates were removed and ANGSD was re‐run as described above. All bioinformatic commands used for quality control and mapping can be found at https://github.com/DeWitP/Mytilus.

### 2.6 Large‐scale population genomic clustering / admixture filtering

Probabilistic genotype estimation was performed using ANGSD version 0.933, filtering out loci with single nucleotide polymorphism (SNP) *p*‐values, strand bias *p*‐values, and heterozygote bias *p*‐values <10^−5^. Also, loci with no mapped reads in less than 50% of the individuals and with a minor allele count less than 5 were discarded. An IBS matrix was generated with the ‐doIBS 1 option in ANGSD, which was hierarchically clustered in R and used to assess the similarity of technical replicates and also to identify potentially closely related individuals. A minimum IBS distance of 0.15 was identified as a useful threshold for filtering out replicates/relatives, below which only one representative individual per cluster was kept for further analysis. The genotyping was then repeated as above without replicates and highly related individuals (*N*_ind_ = 582). The genotype probabilities (*N*_loci_ = 86,375) were analyzed using PCAngsd (http://www.popgen.dk/software/index.php/PCAngsd; Meisner & Albrechtsen (2018)) in order to estimate the most probable number of admixture clusters in the dataset, and individual admixture proportions. Admixture coefficients were plotted using R, and individuals were assigned to one or more admixture cluster(s) using a minimum coefficient threshold of 0.25.

### 2.7 Gene flow patterns in *M. edulis* in the Skagerrak

Genetic differences among the sampling locations in the *M. edulis* lineage dominant in the Skagerrak were investigated using 318 individuals passing all quality control steps and identified as only belonging to the Skagerrak lineage from the admixture coefficients as described above (Table S1). Individuals from the Kattegat were not included in the gene flow estimation, as this area is a known hybrid zone, with introgression with the highly divergent *M. trossulus* DNA (Väinölä & Strelkov, 2011). The genotype probabilities of individuals in loci passing filters (*N*_loci_ = 62,223) were then fed to PCAngsd to generate a SNP covariance matrix and to estimate admixture coefficients and the optimal number of clusters in the dataset. The SNP covariance matrix was used as a distance measure (1‐SNPcov) for multi‐dimensional scaling analyses using the vegan package in R. Variances explained by geographic sampling location and sequencing depth (estimated by the fraction of loci with sequencing depth ≥5 reads) were estimated using PERMANOVA through the adonis R package. The effect of sequencing depth differences among individuals was corrected for using partial ordination, after which the loadings on MDS axis 1 were tested for differences among geographic locations using ANOVA as well as Tukey's HSD test. In addition, as introgression levels varied somewhat in individuals that passed the 0.25 admixture coefficient filter across sampling locations, a regression analysis was performed examining the effect of introgression (admixture proportion of Skagerrak *M. edulis*) on loadings on MDS axis 1.

To infer patterns of gene flow within the *M. edulis* (Skagerrak) lineage, genetic variation was geographically extrapolated using the “effective migration surfaces” (EEMS) method (Petkova et al., 2016). This method places genetic data collection points in a geographic grid pattern of size given by the “nDemes” parameter and then estimates deviation from a general isolation‐by‐distance pattern in order to infer barriers or corridors of gene flow. A geographic polygon defining the Skagerrak area (Table S2) was extracted from Google Earth, after which EEMS was run in three separate runs with different random starting points, using following parameters: 2 million (M) iterations burn‐in, 10 M iterations run length, sampling every 10,000 iterations, nDemes = 300. The results of the three runs were summarized, examined for convergence, and plotted using the rEEMSplots R package (Petkova et al., 2016). In order to examine the robustness of the EEMS output to sampling bias, the software was re‐run with iterative removal of sites 18, 24, and 29 (chosen due to their proximity to inferred barriers). R scripts used for admixture and MDS plotting, as well commands used for the EEMS analysis, can be found at: https://github.com/DeWitP/Mytilus.

## 3 RESULTS

### 3.1 Modeling of larval dispersal and connectivity

**FIGURE 2:**
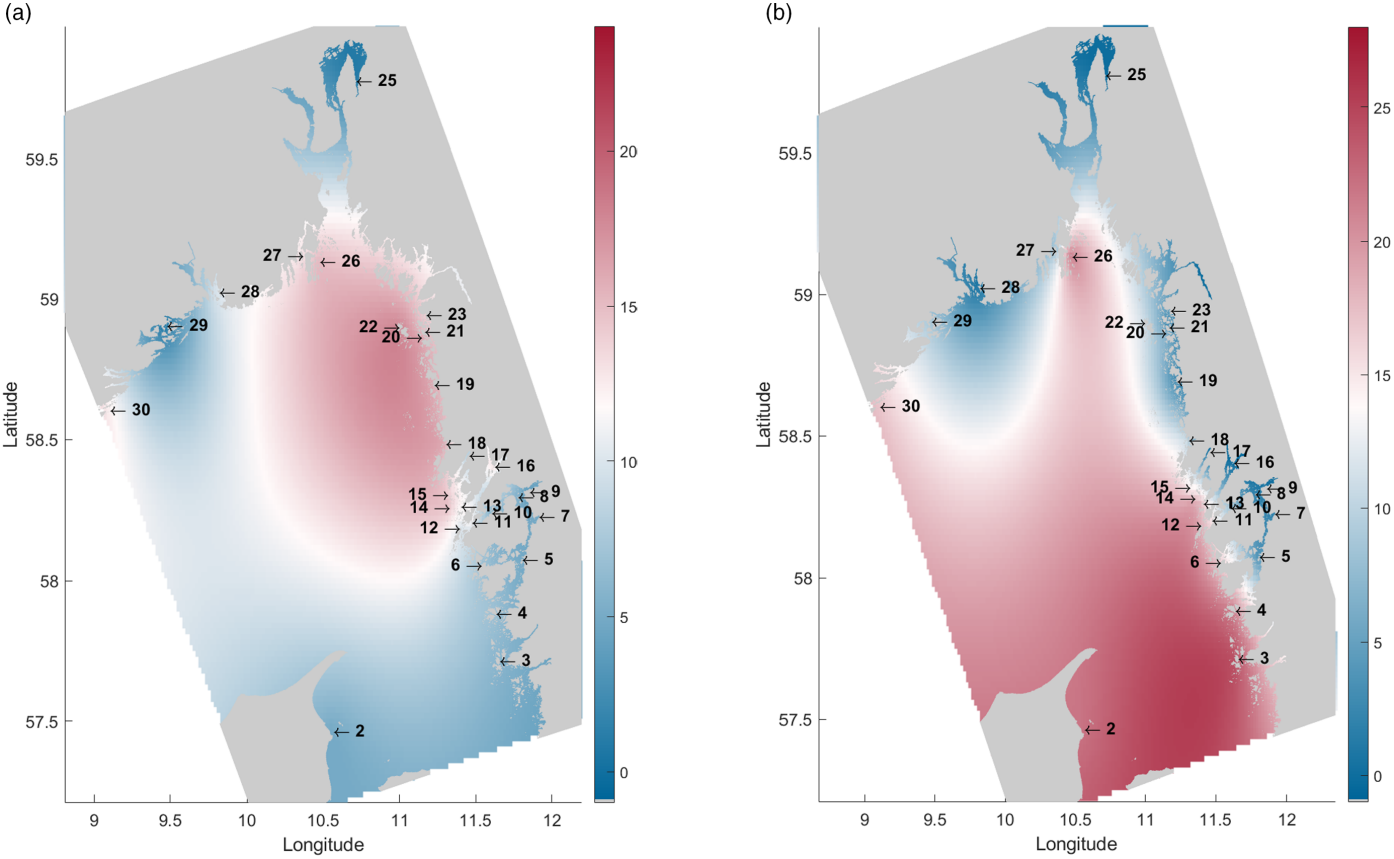
Interpolated larval contributions, based on connectivity among locations, in percent. (a) Sink regions, red colors indicate important sink regions. (b) Source regions, red colors indicate important source regions. Regions with blue color in both A and B indicate areas of low connectivity.

The dispersal and connectivity calculations revealed distinct patterns wherein most sites received larvae from sites located to the south and donated larvae to sites located to the north (Table 2 and Figure 2). The modeling results are presented as a color‐coded connectivity matrix among the study sites (Table 2). The matrix displays the proportion of larvae that moved from one location to another, expressed as a fraction of the total number of released/received larvae. The matrix highlights several key observations. Sites in the southern part of the Skagerrak, particularly those situated in the outer part of the archipelago, such as sites 2–4, contributed larvae to many other sites (21–24), but received a limited number of larvae from just 5–7 other sites. Sites located in the inner archipelago (Table S1) tended to be more isolated, with site 25 (Inner Oslo fjord) being the most isolated with nearly no exchange of larvae with other sites. Sites 8–9, located inside the island of Orust (Table 2) were also relatively isolated. These sites exchanged larvae with each other but received very low input of larvae from the outside (only a small number of larvae from sites 4, 5, and 10; Table 2). Further insight from the results indicated that site 19 (Grebbestad) retained a high proportion of larvae and exported few larvae to other sites, although it received larvae from a high number of sites. Site 28 (Grenland fjord) on the Norwegian coast contributed only very few larvae to the adjacent site 27 (Vrengen), while it received larvae from 14 other sites; the opposite pattern was observed for site 29 (Kragerø), which only received a small number of larvae from a few other locations, but contributed larvae to several sites.

**TABLE 2:** Proportion of larvae transported to (columns) and from (rows) the modeled sites in the study area. Darker red shading indicates higher proportions. Gray shaded boxes indicate local retention of larvae.

	Receiving site																												
	2. Fredrikshavn	3. Öckerö	4. Marstrand	5. Stenungsund	6. Stigfjorden	7. Ljungskile	8. Brattön	9. Uddevalla	10. Hjältön	11. Skaftö	12. Jonsborg	13. Gullmar fjord S	14. Gullmar fjord N	15. Tån	16. Gårvik	17. Åbyfjorden	18. Bovallstrand	19. Grebbestad	20. Svallhagen	21. Tjärnö archipelago	22. Koster	23. Strömstad	25. Inner Oslofjord	26. Færder	27. Vrengen	28. Grenland fjord	29. Kragerø	30. Tvedestrand	Number of sites that each site donated larvae to
2. Fredrikshavn	0.04	0.05	0.06	0.30	0.47	0.07			0.01	0.30	0.35	0.15	0.23	0.37	0.20		1.48	1.42	0.73	0.14	0.38	0.04		0.17	0.30	0.03		0.24	22
3. Öckerö	0.01	0.77	0.51	7.21	1.35	0.54	0.02		0.03	0.40	0.93	0.23	0.31	0.34	0.33	0.10	0.62	0.63	0.27	0.05	0.04	0.02		0.01	0.05	0.03		0.12	24
4. Marstrand		0.04	0.19	22.05	1.14	3.51		0.04	0.01	0.30	0.71	0.13	0.22	0.23	0.40		0.65	0.20	0.12	0.03	0.08	0.03		0.03				0.03	20
5. Stenungsund				25.34		18.37	0.10	0.13					0.01																4
6. Stigfjorden	0.03				19.48				0.03	0.23	0.81	0.12	0.17	0.12	0.32	0.03	0.13	0.27	0.07	0.03	0.06			0.03				0.04	16
7. Ljungskile						66.43	0.03						0.01																2
8. Brattön						0.03	39.34	14.86																					2
9. Uddevalla							10.65	5.36																					1
10. Hjältön							6.28	0.04	10.54	0.11	1.80	0.01	0.06	0.06	0.20		0.04												9
11. Skaftö							0.69		8.00	1.13	6.40	0.26	0.70	0.75	1.65	0.03	0.44	0.06	0.03		0.09			0.07	0.06			0.03	15
12. Jonsborg					0.03				0.37	2.12	0.47	0.16	1.14	1.32	7.00	0.38	1.77	0.67	0.11	0.03	0.02	0.02		0.04	0.08	0.03		0.03	18
13. Gullmar fjord S	0.02	0.01								0.52	0.02	0.09	0.31	1.57	4.63	1.00	2.09	0.76	0.26	0.10	0.13	0.07		0.05	0.09	0.06		0.09	18
14. Gullmar fjord N	0.04	0.01	0.01							0.67	0.03	0.08	0.37	1.67	6.17	0.53	0.94	0.39	0.10	0.04	0.09	0.05		0.03	0.06	0.03		0.03	19
15. Tån			0.03							0.39		0.03	0.21	1.74	0.83	2.73	2.58	0.95	0.25	0.18	0.10	0.08		0.10	0.11			0.14	15
16. Gårvik															36.10														0
.17. Åbyfjorden																73.39													0
18. Bovallstrand					0.03									0.03	0.03		44.73	0.59	0.23	0.03	0.04	0.05		0.04	0.03			0.08	11
19. Grebbestad																		69.88		0.05	0.04	0.02							3
20. Svallhagen																	0.03	0.39	7.36	60.29	0.06	0.35		0.01	0.07				7
21. Tjärnö archipelago																			1.49	74.18	0.08	1.21							3
22. Koster					0.02					0.03			0.03				0.07	0.45	2.29	0.14	24.41	1.11		0.21	0.22	0.07			11
23. Strömstad																		0.19	1.33	7.63	0.08	54.16						0.03	5
25. Inner Oslofjord																							1.28						0
26. Færder	0.01	0.01	0.02	0.03						0.05	0.02	0.04	0.05	0.10	0.03		0.20	0.25	0.19	0.02	0.24	0.06	0.03	0.55	2.93	0.30	0.03	0.31	21
27. Vrengen			0.03									0.03	0.03	0.03			0.07				0.06			1.60	29.42	0.03		0.09	9
28. Grenland fjord																									0.03	11.54			1
29. Kragerø					0.02									0.01			0.07	0.02	0.03		0.03			0.01		0.08	7.97	0.46	9
30. Tvedestrand		0.01	0.01	0.03						0.01	0.03	0.06	0.04	0.05	0.10		0.50	0.36	0.19	0.07	0.14	0.01		0.02	0.04	0.13		5.82	18
Number of sites from which larvae are received	5	6	7	5	7	5	6	4	6	12	10	12	15	14	13	7	16	16	16	15	18	14	1	15	13	10	1	14	

To identify areas of low connectivity, which could be compared with the results from the genetic barrier analysis, the connectivity between locations was interpolated for the entire model domain (Figure 2). Some areas (the innermost part of the Oslo fjord, the area inside Tjörn and Orust, and the area around Kragerø in Norway, blue areas in Figure 2a,b) were found to be isolated and displayed low connectivity to other areas. These locations did not receive many larvae from, or contributed many larvae to, other areas. In contrast, some other areas displayed high connectivity and could be identified as either sink areas (one area in the northeastern part of the modeling domain, the Koster Sea, and the area around Grebbestad and Tjärnö, red area in Figure 2a) which received a large contribution of larvae from various locations, or as source areas (one area in the southeastern part of the domain, southwest of Tjörn, shown as red area in Figure 2b) which contributed most larvae to other areas.

**FIGURE 3:**
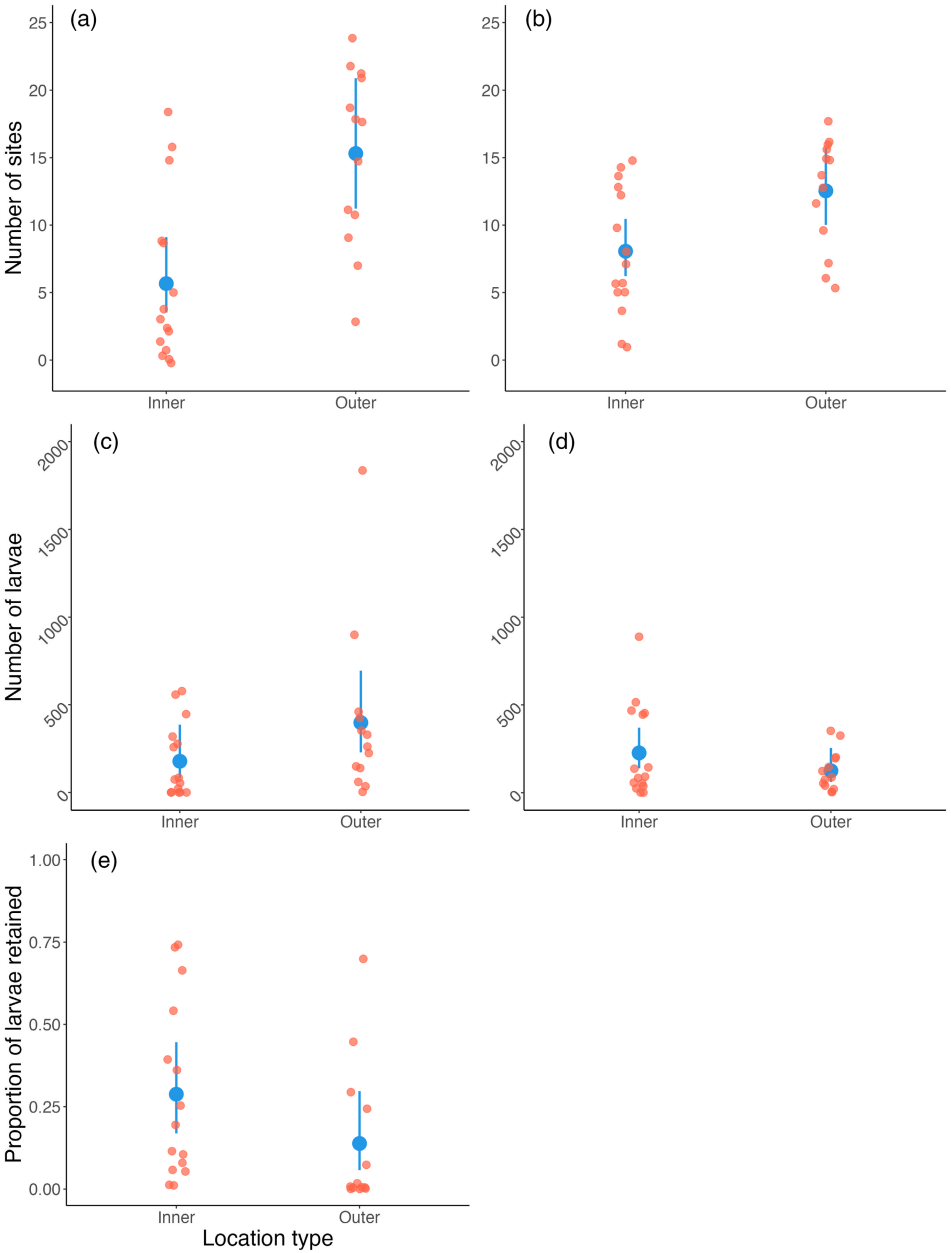
*Mytilus* spp. larval dispersal along the Swedish west coast as a function of site placement in the inner or outer archipelago. The average number of sites that each site contributed larvae to (a) and received larvae from (b), the total number of larvae that each site contributed to (c) and received from other sites (d), and the proportion of larvae that remained at the location of origin (e). All panels show the predictions and their standard errors from generalized linear models (blue) and corresponding raw data (output from oceanographic simulations; red).

The number of sites that each site received from and contributed larvae to were compared based on archipelagic location of the sites (classified as “outer” or “inner” archipelago, *n* = 13 and 15, respectively). The results showed that sites in the outer archipelago contributed with larvae to more sites than did the locations in the inner archipelago (0.994 ± 0.289, *t* = 3.44, *p* = 0.002, Figure 3a), but on average there was no difference between inner and outer locations in how many larvae they provided to other sites (0.803 ± 0.486, *t* = 1.65, *p* = 0.111, Figure 3c). Outer‐archipelago locations received larvae from more sites compared to sites in the inner archipelago (0.441 ± 0.175, *t* = 2.52, *p* = 0.018, Figure 3b), but there were no differences in number of larvae received between inner and outer archipelago (−0.596 ± 0.441, *t* = −1.35, *p* = 0.188, Figure 3d). Finally, there was no difference between inner and outer archipelago in retention of larvae (−0.923 ± 0.606, *t* = −1.52, *p* = 0.14, Figure 3e).

### 3.2 Genetics

#### 3.2.1 Quality control and mapping rates

A total of 602 2b‐RAD libraries were prepared and mapped to the *M. galloprovincialis* genome reference, with a mean of 21.6% (±2.55% SD) of the reads mapping uniquely to one position in the genome (Table S3). Examining the sequence coverage of 2b‐RAD loci in the first 10 scaffolds of the genome assembly, a mean of 81.4% (±8.51% SD) of the loci had a depth of >5 reads (Table S3). Four individuals (NOR‐340, SWE‐033, SWE‐296, and SWE‐319) were discarded due to poor coverage. The IBS control could correctly identify all technical replicates and in addition flagged several individuals as being close relatives. A total of 18 individuals were pruned due to being replicates or closely related, leaving 580 well‐sequenced individuals for further analysis. The two raw data files from the individual used to construct the *M. galloprovincialis* genome assembly (ERR2715051; Gerdol et al., 2020) both had unique mapping rates of 38%, and a sequencing depth >5 covering 22.1% and 18.4% of 2b‐RAD loci in the first 10 assembly scaffolds, respectively. Sequencing depth was generally lower for individuals from site 1 (in the Baltic Sea), likely due to the higher level of genetic divergence between *M. trossulus* and the reference *M. galloprovincialis* genome assembly.

#### 3.2.2 Large‐scale population genomic clustering / admixture filtering

**FIGURE 4:**
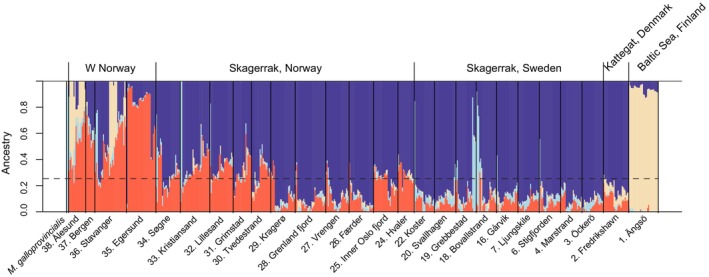
Admixture plot using the optimal K (=4) for *Mytilus* spp. in Scandinavia, ordered by geographic location left—right from the northwest to the southeast. The *Mytilus galloprovincialis* genome individual is included as a reference on the left (light blue). Baltic Sea *M. trossulus* from Ängsö, Finland, are included on the right (yellow). The dark blue and red indicate the Skagerrak and the west Norwegian *M. edulis* lineages, respectively. The dotted line indicates the 25% filtering cutoff, all individuals with more non‐Skagerrak *M. edulis* DNA than this were removed from downstream analyses.

Probabilistic genotyping of the 580 2b‐RAD sequenced individuals plus the two Whole‐Genome Shotgun libraries from the genome assembly individual resulted in 86,375 loci passing all filters. The principal components‐based admixture analysis using PCAngsd indicated the most likely number of clusters (*K*) to be 4. The admixture plot (Figure 4) includes *M. galloprovincialis* (genome assembly individual, light blue bars) and *M. trossulus* (Baltic Sea, yellow bars) references. In addition to *M. galloprovincialis* being introgressed in mussels from the Norwegian west coast, there were also individuals found in Bovallstrand, Grebbestad, Koster, Kristiansand, and Søgne (sites 18, 19, 22, 33, and 34) in the Skagerrak in which a majority of *M. galloprovincialis* ancestry was present. However, it is important to mention that the reference individual used (from Gerdol et al., 2020) may also contain introgressed *M. edulis* DNA (see Materials and Methods), introducing some uncertainty into these estimates. Moreover, *M. trossulus* was found to be introgressed in relatively high proportions (up to >60%) in Stavanger and Ålesund (sites 36 and 38), but not in Egersund and Bergen (sites 35 and 37), also located on the Norwegian west coast. Kattegat (Frederikshavn; site 2) mussels contained ca. 10% *M. trossulus* genetic material, whereas mussels found throughout the Skagerrak consisted only of very small proportions of *M. trossulus* material. Apart from the two clusters with *M. trossulus* and *M. galloprovincialis* reference individuals, the dataset were divided into two separate admixture clusters, red and dark blue in Figure 4, which represented most of the genetic material in mussels from the study area. We will hereafter refer to these two clusters as *M. edulis* (North Sea) and *M. edulis* (Skagerrak). The mean admixture levels between these two clusters were higher than those of the two non‐*M. edulis* clusters, and were particularly high on the southern part of the Norwegian Skagerrak coast and in the Oslo fjord area. For fine‐scale dispersal mapping, mussels which were identified as belonging to the *M. edulis* (Skagerrak) lineage with minimal introgression from other lineages (< 25%, dotted line in Figure 4; *n* = 318) were selected.

#### 3.2.3 Gene flow patterns in *M. edulis* in the Skagerrak

**FIGURE 5:**
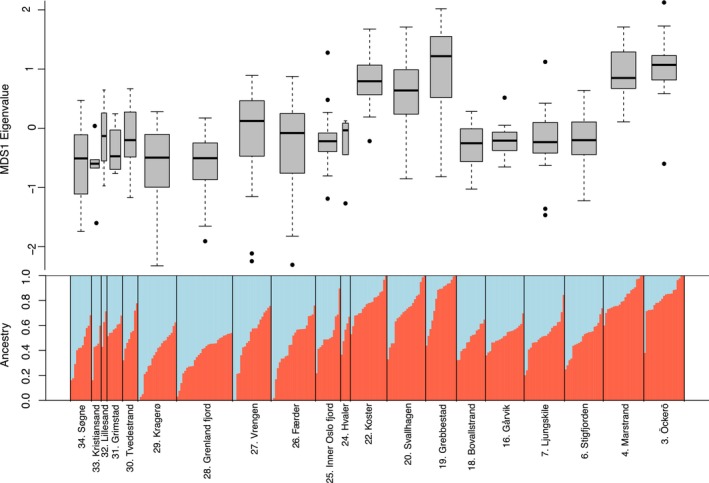
Population genetic patterns within the Skagerrak *Mytilus edulis* lineage, using the SNP covariance matrix. Bottom: Admixture plot of optimal K (=2) determined by pcangsd, sorted by admixture coefficient within each sampling location. Above: Boxplots of MDS1 eigenvalues (explaining 2.35% of the genetic variance).

Both the admixture (above) and MDS analyses (Figure 5) identified two separate genetic entities in the Skagerrak lineage of *M. edulis* (the SNP covariance matrix used for the analyses is in Table S4). The northern (Bovallstrand, Svallhagen, Koster; sites 18, 20, 22) and southern (Öckerö, Marstrand; sites 3, 4) part of the Swedish coast were distinct from all other sampling sites by a larger proportion of the red cluster in the admixture analysis (Figure 5). Although there was much overlap among sites in the MDS analysis (Figure 5, top), MDS axis 1, which explained 2.35% of the variance in the dataset, clearly separated these five Swedish sites from the others. Notably, these five sites were all classified as “outer archipelago” sites. The regression analysis showed a non‐significant effect of introgression of non‐Skagerrak *M. edulis* DNA on the MDS axis 1 loading (*p* = 0.1; Figure S5), indicating that the admixture and MDS analyses were robust to small‐scale differences in introgression.

**FIGURE 6:**
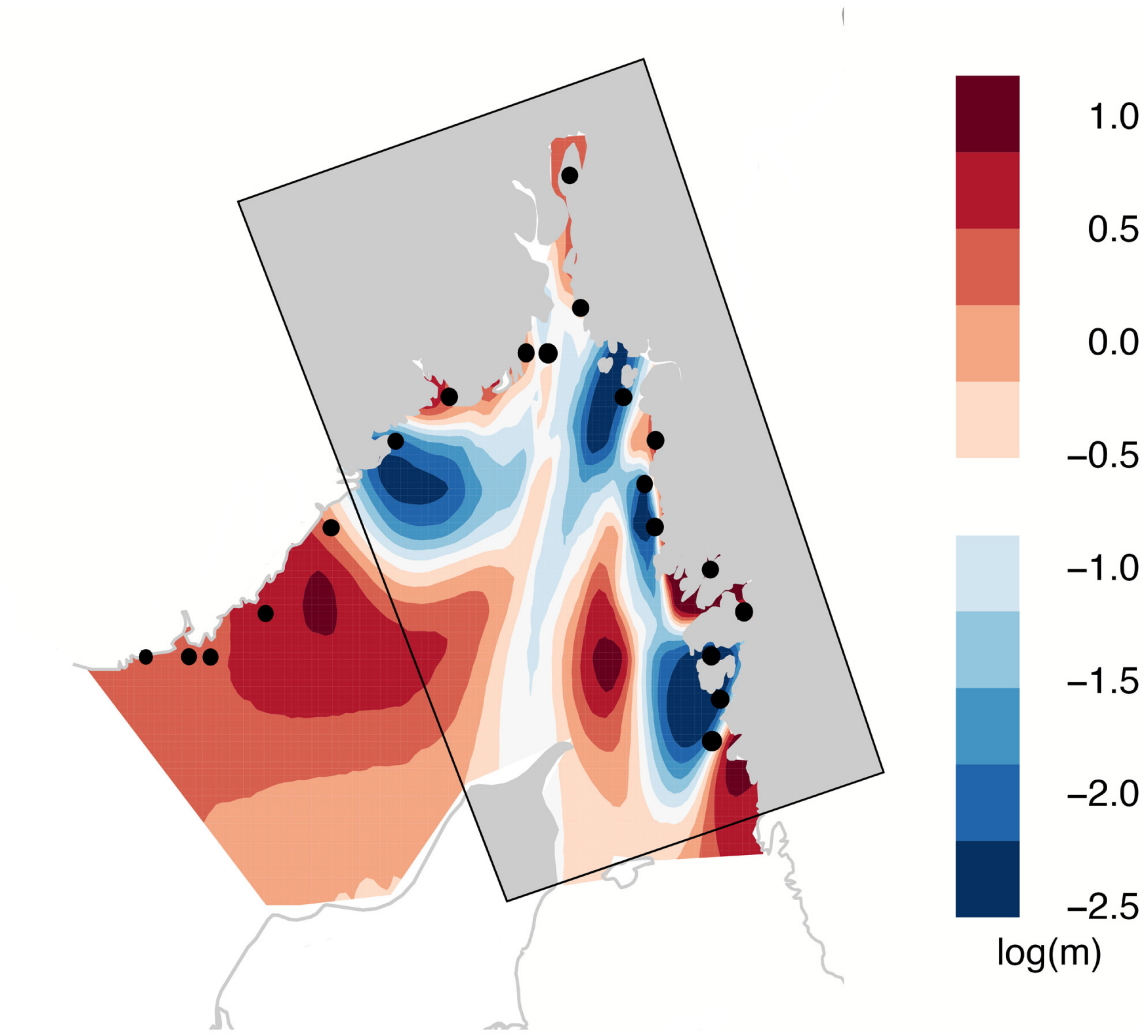
Plot of interpolated barriers to gene flow in Skagerrak (in blue), based on genetic distances among individuals sampled at 21 sites along the Swedish and Norwegian coasts.

The EEMS analysis indicated that there are several barriers to gene flow in the Skagerrak region (blue areas in Figure 6). In particular, sampling locations in the outer archipelago of the west coast of Sweden were separated by two barriers from both the inner fjord/bay areas around Orust and Tjörn islands (the “8 + fjord area,” an ecosystem‐based management pilot area) as well as the Gullmar fjord, respectively. Another barrier, located at the Swedish‐Norwegian border, effectively isolated the Oslo fjord. Finally, a third border was found along the Norwegian coast, in the vicinity of the Kragerø sampling site.

## 4 DISCUSSION

Both the oceanographic modeling and the genomic results display a complex pattern of source‐sink dynamics with several dispersal barriers creating the potential for isolated populations of *M. edulis* in the coastal archipelagos of the Skagerrak.

In our observations, sites located in the southern portion of the oceanographic modeling domain tended to act as source populations, supplying larvae to many sites except to the fjord areas. Conversely, sites in the northern region tended to act as sinks within the modeling domain, primarily receiving larvae (Figure 2). This source‐sink pattern is probably due to the Baltic current providing a prevailing transport along the Swedish west coast from south to north, although it cannot be excluded that the northern region can act as a source for areas outside of the modeling domain. In contrast, sites in the inner archipelago exhibited notable local retention of larvae in the transport model. This difference in retention is likely due to the fact that the currents are stronger, and the water is more open and deeper in the outer archipelago, which favors dispersal, while the long and narrow bays and fjords between the numerous islands along the coastline would favor local retention of larvae, especially in the absence of strong tides (Sponaugle et al., 2002). It must be stated that the hydrodynamical model has apparent weaknesses in that it does not resolve coastline and fine‐scale bathymetry perfectly. Using an ocean current model with 160 m resolution for the Skagerrak is a trade‐off in relation to computing power and precision. However, although the resolution may look limiting, similar model simulations in Norwegian fjords have demonstrated that they can provide realistic estimates of water transports also in narrower waters (e.g., Dalsøren et al., 2020; Simonsen et al., 2023).

The genetic data supported widespread connectivity along the outer archipelago, with very small genetic differences among outer archipelago sites along the Swedish coast. Moreover, the outer archipelago sites seem to receive little larval input from the inner archipelago sites, as evidenced both by the clear genetic differences between sites categorized as “inner” and “outer” archipelago (Figure 6), as well as the oceanographic connectivity analysis (Figure 2). While some of the commonly used population genetic methods have recently been criticized (Elhaik, 2022), we here draw our conclusions from multiple different analysis methods (PCA, NGSadmix, EEMS) indicating the same patterns. We also show data that introgression from other mussel lineages does not seem to have affected the results (Figure S5), which otherwise could be a concern. The high dispersal of larvae from the mussel beds in source areas in the south to more northern mussel beds infers a high importance of these southern mussel beds to the population dynamics of the entire Skagerrak. Recent monitoring data, however, indicates low occurrences of mussels in this specific area (Laugen et al., 2023; Miljöförvaltningen Göteborgs Stad, 2019a, 2019b, 2019c, 2020a, 2020b, 2020c) although the current distribution of mussels is, in general (except for the area around Gothenburg), poorly explored. The lack of knowledge is particularly alarming given the recent reports of mussel populations along the Swedish west coast being in regression (Baden et al., 2021; Laugen et al., 2023). The reasons for the decline of wild mussels are not fully understood. Potential explanations include climate change effects (Baden et al., 2021), or increased predation from, e.g., Eider ducks (*Somateria mollissima*) and European green crabs (*Carcinus maenas*). There is also a lack of knowledge concerning the effects of predation during the pelagic larval phases (Moksnes et al., 2014). Consequently, mapping of mussel occurrences and hotspots in the source population area is essential to assess population status in the area and allow identification of target areas for protection of mussels and management measures such as population enhancement and restoration.

The biophysical transport model and the genetic analyses, while relying on different data, infer similar spatial patterns of connectivity, suggesting that ocean currents are the main driver of dispersal for mussels in Skagerrak. Both the biophysical transport model and the genetic data show that the “8 + fjord” area around the islands of Tjörn and Orust (Cardinale et al., 2017) is isolated from incoming larvae and also exhibits a high degree of local retention. This isolation makes these fjords and bays potentially more sensitive to anthropogenic stressors, as they support the highest concentration of mussel aquaculture on the Swedish coast. In fact, the number of mussels in culture is at least equivalent to, if not greater than, the number of mussels in wild populations in the area (Lindegarth et al., 2019), which suggests a potential large interaction between wild and farmed mussels. Increased predation pressure might be part of the explanation, as mussel farms near wild mussel populations may act as foraging hotspots for Eider ducks (*S. mollissima*) (Kirk et al., 2007; Lindegarth et al., 2019). However, the decline observed in wild mussel populations has not been observed in mussel farms in the area to date, indicating that perhaps the pressure could be due to benthic predators such as crabs or sea stars, from which mussels suspended in farms would be protected.

Moreover, larval behavior, such as settlement preference on anthropogenic substrates (e.g., harbors, boat hulls, mussel farm ropes), may be partly controlled by a genetic component. This was a process we were not able to include in the connectivity modeling, but which has been shown previously in both mussels in Brittany, France (Simon et al., 2019) and in barnacles (Wrange et al., 2016). If this is the case, then anthropogenic selection for attachment to artificial substrates in farms might affect the overall ability of the mussel population to settle on natural substrates. Whether farmed mussels in the study area have evolved to preferentially settle on artificial substrates, and if this impacts the settlement success of larvae on natural substrates, should be explored further to evaluate the potential impact of mussel farming on wild populations. In either case, future restoration efforts of wild stocks in areas with limited larval dispersal should take the isolation into account by using locally produced juveniles and/or brood stock to preserve genetic diversity and potential local adaptation.

Both biophysical modeling and genetics indicate barriers to dispersal in the entrance to the Oslo fjord and in the vicinity of Kragerø along the Norwegian coast. The Skagerrak in general is characterized by a counterclockwise gyre system, with the Baltic Current driving water northward along the Swedish coast in the east, and then turning following the Norwegian coastline southwest. This turn of the current, in combination with the low tidal influence in the area, tends to prevent large‐scale mixing of the Oslo fjord, particularly for surface waters, effectively isolating it from larval input (Anglès d'Auriac et al., 2017). Along the Norwegian coast, near Kragerø, the ocean model indicated that the surface water masses were mainly pushed offshore and into the central Skagerrak more than flowing inshore, which might explain the observed gene flow barrier in this area. Potentially, this could be explained by the bathymetry of the area, with a long string of islands connected by shallow waters. Strings of islands or headlands cause the along‐shore currents to form eddies and vortices which veer offshore, effectively creating a dispersal “shadow” behind them (Sponaugle et al., 2002). In addition, the Norwegian Coastal Current initiated in the inner Skagerrak accelerates west of the Oslo fjord entrance (Sætre, 2007) which could reduce the connectivity along the coast.

The *Mytilus* species complex has a history of multiple separation events followed by secondary contact and introgression in combination with local adaptation patterns, shaping its present‐day complicated genetic patterns (Riginos & Cunningham, 2005; Touchard et al., 2023; Wenne et al., 2020). *Mytilus edulis* in Europe has been shown to consist of a southern and a northern European lineage, where the northern lineage is characterized by introgression from the North American *M. edulis* line (Kijewski et al., 2019). In the most comprehensive study of *Mytilus* patterns in the North Atlantic to date, Wenne et al. (2020) found introgression of North American *M. edulis* at a site (SAL) that is only a few km from our sampling site 20 (Svallhagen), similar to the introgression observed on the Norwegian north‐west coast (Bodø and Tromsø). In contrast, we found that the Skagerrak mussels in most sampling locations (dark blue in Figure 4) were distinct from the ones found on the Norwegian west coast, suggesting that they might belong to the southern lineage. However, as we did not include any samples from southern Europe in our analysis, nor any reference for North American *M. edulis*, we cannot make any strong conclusions at present. In the Kattegat, the introgression of *M. trossulus* alleles is prevalent, and although *M. trossulus* alleles are relatively few in the northern Kattegat as observed in the overall admixture analysis (Frederikshavn data (site 2) in Figure 4), the genetic composition is highly divergent from that of *M. edulis*, swamping the signal of gene flow within *M. edulis* if included in the analysis (data not shown). The Baltic *M. trossulus* lineage that we here have used as a reference for the species is known to consist of a “hybrid swarm,” with wide‐scale introgression of low amounts of *M. edulis* DNA (Riginos & Cunningham, 2005), which might have skewed our admixture analyses of introgression in the Skagerrak. However, the level of *M. trossulus* introgression we observe in our Kattegat site is similar to what has previously been reported (Stuckas et al., 2017), supporting our findings. The fine‐scale distribution of *M. trossulus* introgression in the Kattegat hybrid zone has yet to be studied, but would be of great importance to local restoration efforts of mussel beds. The fitness landscape of the different mussel genotypes along the complex shoreline of the Baltic transition zone with a strong salinity gradient is also not well known. There is some evidence that *M. edulis* × *M. trossulus* hybrids could have a higher fitness in low salinities (Michalek et al., 2016), which might make them predisposed to colonize river estuaries in the area. Also, a recent study found that certain combinations of *M. edulis* × *M. galloprovincialis* hybrids seem to successfully colonize multiple ports along the coast of France, suggesting that they might have a fitness advantage in that environment (Touchard et al., 2023). If there is local adaptation within the *M. edulis* lineage to outer versus inner archipelago conditions, or if there is differential selection for larvae to settle on natural vs. artificial substrates (e.g., in mussel farms) has not been studied to our knowledge, but would be highly interesting avenues for further research. However, differences in selective pressures would only be expected to affect parts of the genome, and thus are unlikely to cause the patterns that we observe here, as the 2b‐RAD markers that we have used are randomly spread out through the entire genome.

## 5 CONCLUSIONS AND MANAGEMENT ADVICE

This study sheds light upon the geographical distribution of genetic diversity in blue mussels along the Skagerrak coast. It reveals the existence of small‐scale geographical barriers even for marine species with long pelagic larval duration, which remains poorly documented. The results also provide a good basis for an efficient and constructive local management of blue mussel populations along the Skagerrak coast and are an example of how we can combine genetic and oceanographic tools to refine and detail the patterns observed by each method separately. This has implications in terms of establishment of conservation actions such as restoration, as well as for aquaculture.

From a management perspective, we show that some areas can be isolated despite the overall high dispersal ability of blue mussels. In such situations, the mussel populations along the coastline should be managed as separate subunits as there are extensive genetic structures and dispersal barriers in the area, such as the area inside of Orust and Tjörn which needs special attention in this context. Additionally, considering the low connectivity between the inner and outer archipelago found in this study, preservation of both coastal mussel beds and beds in offshore environments is necessary. Moreover, there is a need to preserve populations located in various geographical areas, including areas that constitute important source areas (e.g. Gothenburg area in this study) or important sink areas where large quantities of larvae gather (e.g. Koster area in this study).

Translocation of mussels between areas should thus be avoided. Mussel beds based on locally recruited mussels should be maintained in all areas, in order to allow for naturally occurring gene flow between populations meanwhile maintaining the genetic diversity within specific geographical areas and ensuring local adaptation potential. Consequently, the population structures and dispersal patterns of an organism should be known before management actions are implemented. In areas where genetically differentiated populations and dispersal barriers exist within a given geographic area, management decisions and strategies should be designed to maintain these structures.

## CONFLICT OF INTEREST STATEMENT

Pierre De Wit is an Editorial Board member of Evolutionary Applications and a co‐author of this article. To minimize bias, they were excluded from all editorial decision‐making related to the acceptance of this article for publication.

## Supporting information


Data S1.

## Data Availability

All raw genetic data produced in this study are available at NCBI, BioProject PRJNA1075926. The SNP covariance matrix used for population genomic inferences is available as supplemental data appended to the manuscript.
