# Supplementary material for: Unlocking the secret life of blue mussels: Exploring connectivity in the Skagerrak through biophysical modeling and population genomics
Source: Evol Appl. 2024 May 20;17(5):e13704. doi: 10.1111/eva.13704 (PMC11104481; doi:10.1111/eva.13704)
Supplement: Supplementary file 1 — Data S1. [file EVA-17-e13704-s001.zip › S5 Suppl Fig S5.pdf]

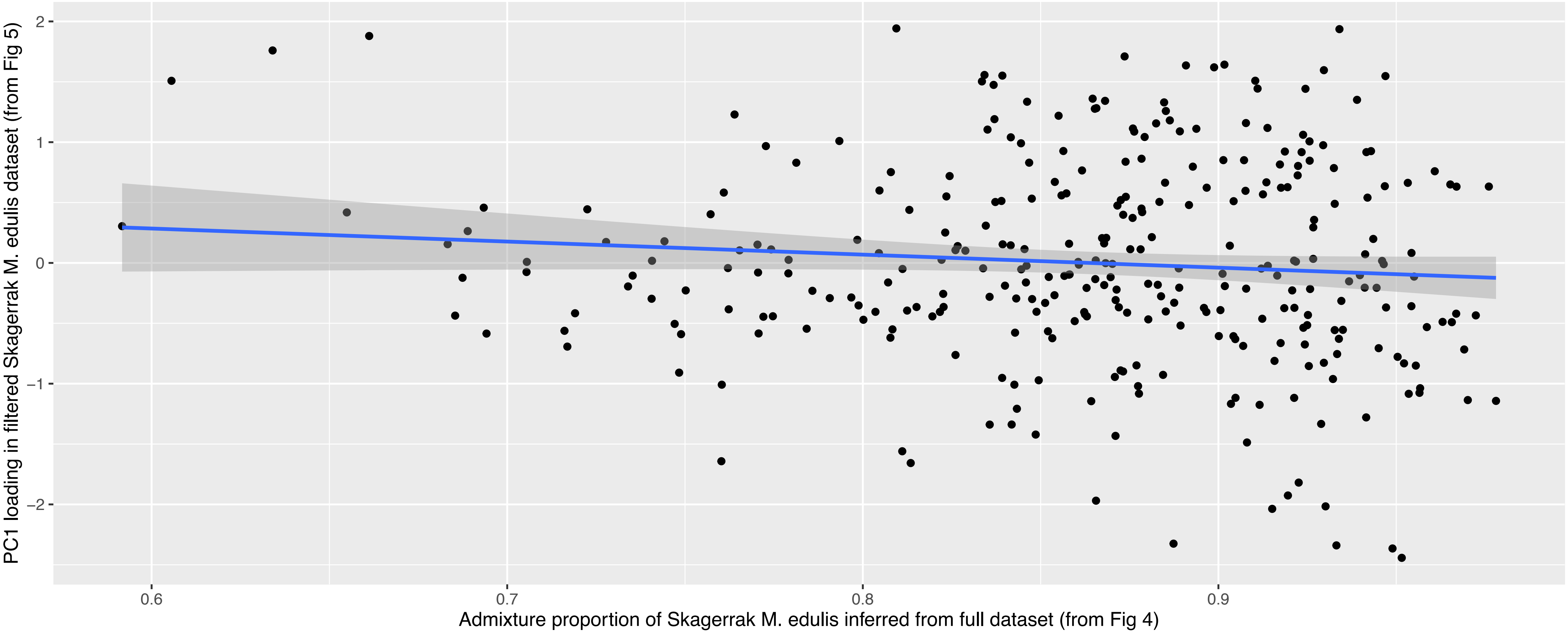

Supplementary Figure S5. Lack of correlation (p = 0.1, r2 = 0.005) between the amount of introgression of non Skagerrak M. edulis DNA and the results of the PCA (eigenvactor 1 loadings) in the dataset filtered to only include Skagerrak M. edulis individuals.
